# Supplementary material for: Next-Generation Sequencing-Based Study of Helicobacter pylori Isolates from Myanmar and Their Susceptibility to Antibiotics
Source: Microorganisms. 2022 Jan 17;10(1):196. doi: 10.3390/microorganisms10010196 (PMC8781859; doi:10.3390/microorganisms10010196)
Supplement: Supplementary file 1 [file microorganisms-10-00196-s001.zip › microorganisms-1554492-supplementary.pdf]

Supplementary Table S1. The selected *H. pylori* strains for genotyping study by whole-genome sequencing.

Patterns	No.	Strains	AMX	CAM	LVX	MNZ	TCN
All sensitive	1	MMM 8	S	S	S	S	S
	2	MMM 23	S	S	S	S	S
	3	MMM 26	S	S	S	S	S
	4	MMM 58	S	S	S	S	S
	5	MMM 90	S	S	S	S	S
Single-drug resistance	6	MMM 44	S	S	R	S	S
	7	MMM 54	S	S	R	S	S
	8	MMM 74	S	S	S	R	S
	9	MMM 84	S	S	S	R	S
	10	MMM 86	S	R	S	S	S
	11	MMM 108	S	S	S	R	S
	12	MMM 137	S	S	S	R	S
Double-drug resistance	13	MMM 3	R	R	S	S	S
	14	MMM 25	R	S	S	R	S
	15	MMM 33	S	S	R	R	S
	16	MMM 37	R	S	R	S	S
	17	MMM 47	S	S	R	R	S
	18	MMM 62	S	S	R	R	S
	19	MMM 135	S	S	R	R	S
	20	MMM 145	S	S	R	R	S
Triple-drug resistance	21	MMM 43	S	R	R	R	S
	22	MMM 131	S	R	R	R	S
	23	MMM 149	S	R	R	R	S

R; Resistance S; Sensitive

Supplementary Table S2. Mutations in *rdxA* and *frxA* that were associated with MNZ-resistance.

Gene	Strain	MMM25	MMM33	MMM43	MMM47	MMM62	MMM74	MM84	MMM108	MMM131	MMM135	MMM137	MMM145	MMM149
	MIC (mg/L)	256	>256	16	96	96	>256	128	16	128	>256	>256	32	24
rdxA	Position		Could not obtain the sequence											
	6										R/H			
	16							R/H	R/H		R/C	R/H		
	56					H/R						H/R		
	65								L/V			L/V	L/V	
	70													A/V
	71						A/V							
	83								A/S					
	91							S/P	S/P		S/P	S/P	S/P	
	97			L/F										
	98			L/V										
	99			P/A										
	116						V/M							
	121				A/F							A/S		
	122				A/L									
	143					C/*								
	170									L/C				
	171									K/*				
	175	V/I		V/I				V/I	V/I		V/I	V/I	V/I	V/I
	196						A/T							
	209	A/T												
	212										W/*			
frxA	19	No mutation detected							No mutation detected		Y/T			
	20										D/I			
	23										R/V			
	24										R/V			
	25										I/F			
	26										S/P			
	27										Q/K			
	28										K/R			
	29										D/I			
	30										W/G			
	31										E/K			
	33		L/M								L/M			L/M
	37									G/R				
	40													A/S
	43											S/A		
	60				K/R	K/R								
	83						Y/S							

	85						A/V							
	98												K/E	
	101												H/M	
	102												E/R	
	103												V/L	
	110											T/A		
	117											I/M		I/M
	154													A/T
	184			A/V							A/M			
	192							A/G						
	193							F/*						

A; Alanine, C; Cysteine, D; Aspartic acid, E; Glutamic acid, F; Phenylalanine, G; Glycine, H; Histidine, I; Isoleucine, K; Lysine, L; Leucine, M; Methionine, P; Proline, Q; Glutamine, R; Arginine, S; Serine, T; Threonine, V; Valine, W; Tryptophan, Y; Tyrosine, *; Translation stop, MMM; bacteria strain

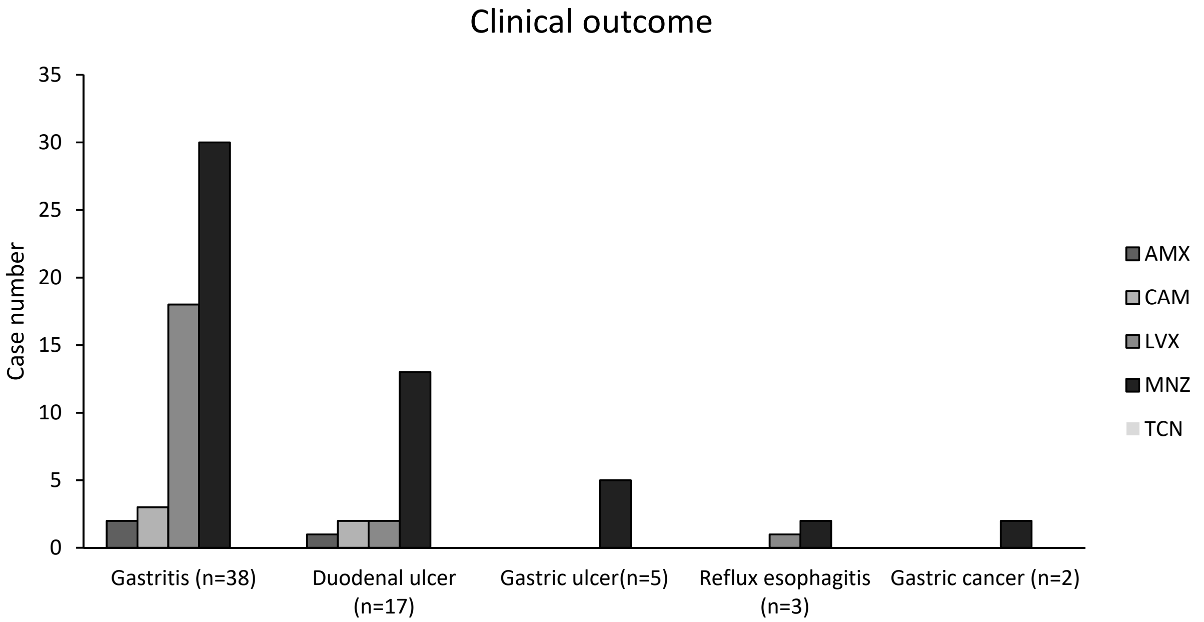

Supplementary Figure S1. Distribution of antibiotic resistance based on the clinical outcome. AMX; amoxicillin, CAM; clarithromycin, LVX; levofloxacin, MNZ; metronidazole, TCN; tetracycline.
